# Common and Unique Network Dynamics in Football Games

**DOI:** 10.1371/journal.pone.0029638

**Published:** 2011-12-28

**Authors:** Yuji Yamamoto, Keiko Yokoyama

**Affiliations:** 1 Research Center of Health, Physical Fitness and Sports, Nagoya University, Furo-cho, Chikusa, Nagoya, Japan; 2 Department of Psychology and Human Developmental Sciences, Nagoya University, Furo-cho, Chikusa, Nagoya, Japan; 3 Division of Applied Physics, Faculty of Engineering, Hokkaido University, Kita-ku, Sapporo, Hokkaido, Japan; 4 Japan Society for the Promotion of Science, 5-3-1, Koujimachi, Chiyoda-ku, Tokyo, Japan; University of Maribor, Slovenia

## Abstract

The sport of football is played between two teams of eleven players each using a spherical ball. Each team strives to score by driving the ball into the opposing goal as the result of skillful interactions among players. Football can be regarded from the network perspective as a competitive relationship between two cooperative networks with a dynamic network topology and dynamic network node. Many complex large-scale networks have been shown to have topological properties in common, based on a small-world network and scale-free network models. However, the human dynamic movement pattern of this network has never been investigated in a real-world setting. Here, we show that the power law in degree distribution emerged in the passing behavior in the 2006 FIFA World Cup Final and an international “A” match in Japan, by describing players as vertices connected by links representing passes. The exponent values 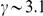 are similar to the typical values that occur in many real-world networks, which are in the range of 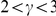, and are larger than that of a gene transcription network, 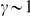. Furthermore, we reveal the stochastically switched dynamics of the hub player throughout the game as a unique feature in football games. It suggests that this feature could result not only in securing vulnerability against intentional attack, but also in a power law for self-organization. Our results suggest common and unique network dynamics of two competitive networks, compared with the large-scale networks that have previously been investigated in numerous works. Our findings may lead to improved resilience and survivability not only in biological networks, but also in communication networks.

## Introduction

Football is played by some 265 million people throughout the world. For many people, the key attraction of the game is its unpredictability (i.e., its emergent properties). All eleven players work together to control the ball under strict rules that require passing in a limited space. From the viewpoint of network topology, this game can be considered to represent the interaction between two competitive and internally cooperative complex networks. A small-world network model [1] and a scale-free network model [2] have been published, and complex large-scale networks such as the World Wide Web [3], social networks [4], and cell biology [5] have been shown to have topological properties in common. Many natural networks contain a few nodes, termed hubs, that have many more connections than the average node does. In this type of network, termed scale-free networks, the fraction of nodes having 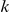 edges, 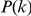, decays according to the power law, 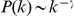. The self-organization of networks frequently coincides with the appearance of power-law distributions. Because these studies have focused on large-scale networks that have at least 100 nodes, it is unclear whether this framework can be applied to networks such as football games. The degree of distribution in the network of a football game could be assumed to show a power law or hubs because football teams have particularly dominant players who tend to control the game. However, vulnerability to an intentional attack is a serious problem in football, although the hub in scale-free networks is resistant to a random attack [6]. It is easy to imagine attacking a hub to gain an advantage over the opponent because the two football teams are in a competitive relationship.

Most of the related studies have been concerned with the dynamics of networks, and the network topology itself is regarded as a dynamic system. Another line of network research focuses on the dynamics *on* networks [7], in which each node of a network represents a dynamic system. Adaptive networks have been investigated from the point of view of dynamical criticality [8], and these networks combine the topological evolution of network topology with dynamics in network nodes [9], [10]. The essential characteristic of adaptive networks is the interplay between dynamics on the network and dynamics of the network, and the feedback loop can give rise to a complex mutual interaction between a time-varying global topology and the local dynamics [10]–[13]. From this viewpoint, the topology of each network, that is, the pattern of cooperation exhibited by each team, influences the local dynamics (e.g., the movement of players), and the dynamics of the players, in turn, affect the team's pattern of cooperation. When the hub is attacked intentionally by the opponent, the topology of the network and the state of the player as a node can be switched. Consequently, the hub can be switched. That is, competition between the teams forces each team to switch its pattern of cooperation to maximize team performance.

Here, we examined common and unique network dynamics in football games as a typical example of competitive relationships between two cooperative networks with a dynamic network topology and local dynamics. This approach may contribute to the development of resiliency in other social networks.

## Results

### Degree distribution

**Figure 1:**
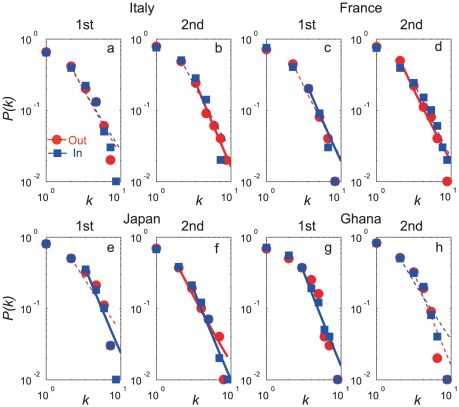
The distribution function of consecutiveness for each outgoing and incoming passing network. **a** and **b**, Outgoing and incoming links for Italy in the first and second halves, respectively. The red and blue lines in panel **a** have slopes 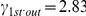 and 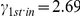, respectively, and those in panel **b** have slopes 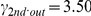, and 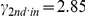, respectively. **c** and **d**, Outgoing and incoming links for France in the first and second halves, respectively. Red and blue lines have slopes 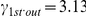, 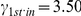, 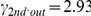, and 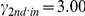, respectively. **e** and **f**, Outgoing and incoming links for Japan in the first and second halves, respectively. Red and blue lines have slopes 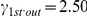, 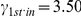, 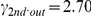, and 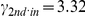. **g** and **h**, Outgoing and incoming links for Ghana in the first and second halves, respectively. Red and blue lines have slopes 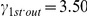, 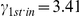, 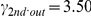, and 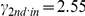. Solid lines show power law distributions, and dashed lines show power law with cut-off distributions (Text S1).

We analyzed the probability distribution for the connectivities of the vertices or the players 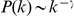 as a means of examining the power-law scaling [2] for inward links and outward links separately. Additionally, we treated the first and second halves separately. We also compared the power-law with alternative hypotheses (see Text S1). The degree distributions followed a power law 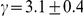, a common feature of large-scale scale-free networks (see Table 1, Table 2, Table S1, Fig. 1). The exponent values 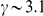 are similar to the typical values that occur in many real-world networks [3]–[5], which are in the range 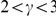. Growth and preferential attachment have been identified as the two basic mechanisms responsible for the scale-free property [2], but the small exponent value 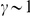 could be explained by a simple rewiring process of links without growth [14], corresponding to scale-free biological networks [15]–[17].

**Table 1:** Power law in degree distribution with respect to outgoing and incoming link for each half and goodness-of-fit tests in World Cup 2006.

			n	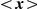	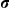	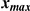	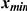	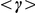	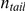	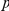
Italy	1st	Out	147	13.4	8.38	29	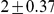	2.83	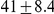	0.08
		In	147	13.4	8.03	31	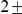 0.67	2.69	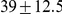	0.01
	2nd	Out	171	15.5	6.83	28	3 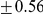	3.50	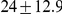	**0.51**
		In	171	15.5	5.31	23	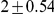	2.85	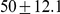	0.00
France	1st	Out	148	13.5	3.23	21	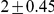	3.13	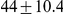	0.05
		In	148	13.5	4.03	22	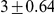	3.50	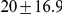	**0.23**
	2nd	Out	173	15.7	6.68	27	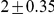	2.93	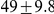	**0.32**
		In	173	15.7	8.38	26	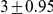	3.00	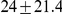	0.09

Each column denoted the number (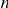), mean (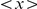), standard deviation (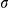), and maximum value (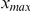) of observations, and the lower cut-off at which the power law no longer applies (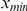), the slope of the power law in the power law region (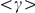), the number of observations in power law region (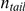), and 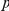-values of the goodness-of-fit tests (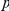).

**Table 2:** Power law in degree distribution with respect to outgoing and incoming link for each half and goodness-of-fit tests in Kirin Cup 2006.

			n	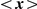	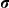	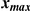	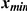	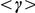	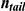	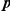
Japan	1st	Out	197	17.9	5.05	24	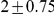	2.50	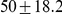	0.00
		In	197	17.9	4.60	27	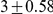	3.50	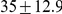	**0.45**
	2nd	Out	151	13.7	6.55	27	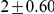	2.70	37 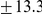	**0.21**
		In	151	13.7	7.62	30	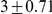	3.32	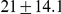	**0.19**
Ghana	1st	Out	205	18.6	11.61	44	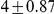	3.50	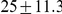	0.03
		In	205	18.6	10.88	40	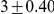	3.41	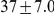	**0.39**
	2nd	Out	197	17.9	8.68	36	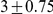	3.50	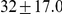	0.04
		In	197	17.9	9.40	35	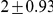	2.55	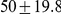	0.00

Each column denoted the number (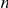), mean (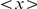), standard deviation (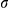), and maximum value (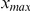) of observations, and the lower cut-off at which the power law no longer applies (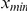), the slope of the power law in the power law region (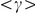), the number of observations in power law region (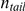), and 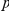-values of the goodness-of-fit tests (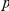).

These results show a feature common to other large-scale networks that contain a few nodes with many more connections than the average node has. These scale-free networks have been shown to have self-organizational and emergent properties, suggesting that the dynamic network topology in football games may also have this property.

### Switching hubs

**Figure 2:**
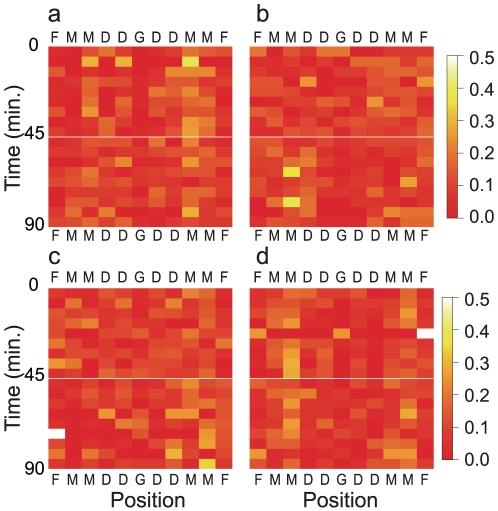
Relative ball touch frequencies for each player in each 5-min interval. The goalkeeper, defender, midfielder, and forward are denoted as G, D, M, and F, respectively. The color gradation from red to white corresponds to an increase in the relative frequencies of ball touch from 0% to 50%. **a**–**d**. Italy, France, Japan, and Ghana, respectively.

For many real-world networks that show exponent values in the range of 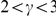 and for biological networks that show 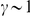 in degree distributions, the hubs are assumed to be static. However, in a network that has only a few vertices, such as a football team, it is necessary to have both low vulnerability against intentional attack and a power law for self-organization of the network. Because both error tolerance and self-organizing properties are required in a football game, it is assumed that the network topology changes to follow the power law when the function of the hubs switches to another vertex. To demonstrate this, Figure 2 shows the frequencies of passing involvement within the same team in 5-min intervals. Although there is no color variation if the hubs have not switched, it is obvious that the player who touches a ball many times has switched to other players in terms of sending and receiving passes. That is to say, a switch in the topology of the network is correlated with the attack by the opposing team and represents an adjustment in the cooperation within each network during competition between networks, which is regarded as the local dynamics. The dynamics of these networks are similar to self-organized criticality examined by a cellular automaton [8] or the ‘Game of Life’ [18], or to spontaneous structure formation in coupled chaotic systems [19], [20]. However, in those cases, only one network exists versus competition between two networks as described here.

These results show a unique feature of the competitive relationship between two cooperative networks. In football games, to gain the advantage over the opponent, dominant players, the hubs, who touch the ball more frequently and tend to control the game would be susceptible to intentional attacks by opponent players.

### Triangles and successful attacks

**Figure 3:**
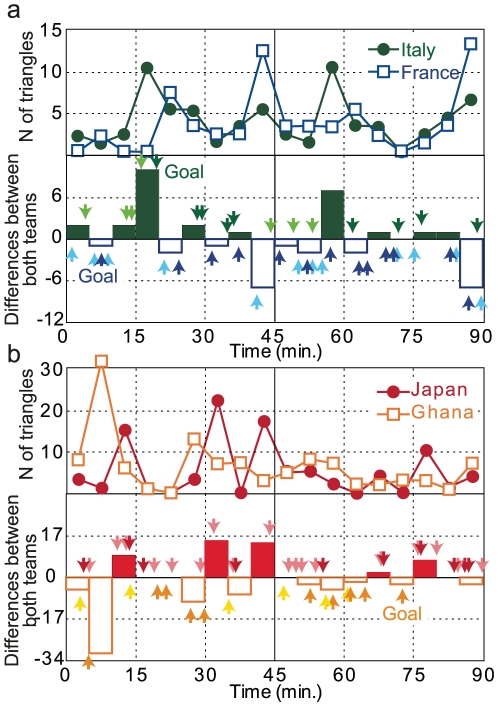
Game momentum, represented by the number of triangles in each network. **a**, Upper panel shows the number of triangles for each team during each 5-min interval of the 2006 World Cup Final. Differences between the numbers of triangles for both teams in each 5-min interval are shown in the lower panel as a bar chart. A green bar shows that the number of triangles was greater for Italy than for France, and a blue bar shows that France generated more triangles than Italy. The attacking phase also includes the shots added to the lower panel as green and blue arrows for Italy and France, respectively. The thick and thin arrows show the shots and the successful attacks without the shots, respectively. **b**, The case of the 2006 Kirin Challenge Cup shown in a manner similar to that for the 2006 World Cup in panel **a**. Fisher's exact tests were applied to determine whether more triangles generated by a team correlated with the more attacks during that time period. The 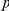-values for this correlation were 0.087 for the World Cup and 0.024 for the Kirin Cup.

To examine competition between the two networks, we counted the triangles formed in each 5-min interval (Fig. 3). The number of triangles is a simple measure of network structure; this measure is the origin of the structural analysis used in the field of social sciences known as modularity [21] or network motifs [22], [23]. We found varying tendencies in these time series in the number of triangles in each 5-min interval during the game, suggesting that a well-organized attack could not continue due to the competition between the two teams. In the World Cup, when Italy generated more triangles in each 5-min interval, Italy had nine successful attacks and France had six. In contrast, when France had more triangles, Italy had six attacks and France had 16. In the Kirin Cup, when Japan generated more triangles, Japan had 10 attacks and Ghana had one, and when Ghana had more triangles, Japan had 11 attacks and Ghana had 12 attacks. Fisher's exact test was used to analyze the relationship between numbers of triangles and the frequency of successful attacks and shots; results showed 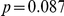 for the World Cup and 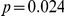 for the Kirin Cup, indicating that the team that formed more triangles obtained more attack opportunities. That is, the team with more triangles, thought to be a basic element of the team network, has an advantage in terms of game momentum even though the hub changed. This finding suggests that the number of triangles may represent the game momentum [24].

## Discussion

We analyzed the probability distribution that emerged in the passing behavior in the 2006 FIFA World Cup Final football game and an international “A” match in Japan by describing players as vertices connected by links representing passes. As a result, we showed that the degree distributions followed a power law with 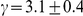. These common features of a dynamic network topology with large-scale networks have been investigated in numerous works. These exponent values, 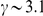, are similar to, are similar to the typical values that occur in many real-world networks [3]–[5], in the range 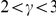, and are larger than that of a gene transcription network [15]–[17], 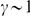.

To understand this exponent value, i.e., 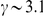, we analyzed the frequencies of passing involvement within the same team in 5-min intervals. The results showed that it was obvious that a player who touched the ball many times changed the player to whom he was connected by passes. Because both error tolerance and self-organizing properties [6] are required in a football game, it is assumed that the network topology changed to follow the power law while the function of the hub switched to another vertex. The local dynamics of the network would be a unique feature in football.

In this case, the overall system of the game consists of two competitive networks. Although these structures have been examined as an example of outer synchronization [25]–[27], they will never, in fact, synchronize because the two networks in a football game have mutually exclusive goals. The two networks are connected by a feedback loop representing competition. From the viewpoint of network B, network A is regarded as external input, and it influences the global topology and local dynamics of network B. Thus, the topology of network B will change based on this competition. This new topology of network B will be regarded as the external input for network A, and a corresponding update will occur in network A. This conception of the system suggests that stochastically switched dynamics with temporal input are necessary to a consideration of the dynamics of each network. The external inputs can be switched stochastically, and the fractal transitions between attractors have been examined theoretically [28], [29] and experimentally [30]. The two competitive networks have properties both as a whole and as parts. The intra-team network is self-organized as a part, and the behavior of the inter-team network is simultaneously self-organized as a whole because the hub is switched by inter-network competition. Considering the system as a whole allows us to regard it as a closed conventional dynamic system, expressed as 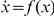. On the other hand, considering each subsystem as a part requires an examination of the stochastically switched dynamics as an open system, expressed as 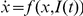, where 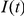 means external input. These networks would be regarded as a hybrid dynamic system [31]. Our findings in football games could provide concepts to improve resilience and survivability not only in biological networks, but also in communication networks.

From another viewpoint, a football game could be regarded as a coevolutionary game, in which coevolutionary rules are introduced, theoretically speaking, to evolutionary game theory [32], [33]. However, it is still an open question as to whether this perspective reveals emergent factors in a football game.

## Methods

We analyzed networks constructed using recorded football matches. Data were obtained from the match between Italy and France in the 2006 FIFA World Cup final in Germany (the teams' FIFA rankings were second and fourth at that time, respectively) and an international “A” match (the 2006 Kirin Challenge Cup) in Japan between Japan and Ghana (with FIFA rankings of 47th and 28th, respectively). According to records kept by the FIFA, the former was 120 min long, including 30 min of extra time, but we analyzed only the first 90 min of the match. The latter was 93 min long, including three extra minutes, and the entire match was analyzed.

**Figure 4:**
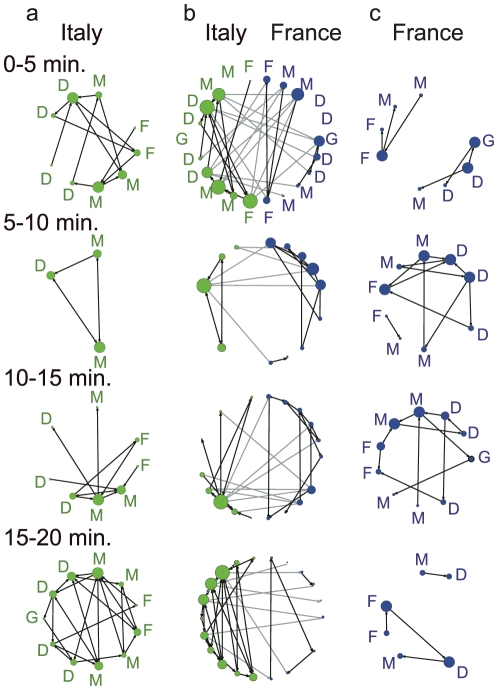
Wiring diagrams for a football game. All diagrams represent each player as a vertex and each pass as a link in each 5-min interval in the first 20 min of the first half. The size of each vertex shows its degree. France received a penalty kick and scored in the seventh minute. Italy scored in the 19th minute after a corner kick. The black line shows the tracking of the ball handling during each 5-min interval for the same team. **a**. Intra-group network for Italy. **b**. Inter-group network for both teams. The gray line also shows the tracking of the ball between teams. **c**. Intra-group network for France. G, D, M, and F denote goalkeeper, defender, midfielder, and forward, respectively, according to the 4-4-2 system of play, regardless of the system actually chosen by each team.

We recorded all changes in possession, not just passes between teammates (see Table S2, Table S3). Passes were recorded in bracketed pairings; in each case, the first number indicates the passing player, and the second number identifies the receiving player. Using I and F to represent Italy and France, respectively, and a single digit to designate each player (rather than the uniform number), passes were recorded as {I6, I9}, {I9, F4}, {F4, I8}, and so on. Then, we described networks for each team by regarding each player as a vertex and each pass as a link. The number of passes per player was counted in 5-min intervals, which we selected as the optimal time frame for the purposes of this study. A football match consists of two 45-min periods; the divisors of 45 are 1, 3, 5, 9, 15, and 45. We concluded that 1- and 3-min intervals would be too brief to encompass a reasonable number of passes, and that 9-, 15-, and 45-min intervals would yield too few segments to allow examination of the momentum in the match. The network was refreshed every 5 min, resulting in 18 different networks throughout the game (see Fig. 4, Fig. S1, Fig. S2). We analyzed the probability distribution for the connectivities of the vertices, or the players, 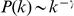 as a means of examining the power-law scaling [2] for inward links and outward links separately. Additionally, we treated the first and second halves of the games separately. In addition, we have evaluated these power-law distributions against other alternatives [34] (see Text S1).

To examine competition between the two networks, we counted the triangles formed in each 5-min interval. When three nodes were connected by two links, we counted them as two triangles. However, when three nodes were connected by one double link and two single links, we counted them as one triangle. Triangles were identified and counted using Combinatorica programming in Mathematica. Also, we characterized the attack phase as attempts to shoot, crosses, dribbling, free kicks, or corner kicks taking place in the attacking third of the field. These categorizations were determined using video replay with the help of the match commentary. Then, these successful attacks were put into two cases, according to which team generated more triangles in each 5-min interval. As a result, a 2 (team) 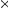 2 (case) frequency table was obtained. To analyze the relationship between the numbers of triangles and the frequency of successful attacks, Fisher's exact test was used.

## Supporting Information

Figure S1 **Network diagram described by players as vertices connected by links representing passes in each 5-minute intervals in World Cup 2006.** Black lines show the passes within each team, gray lines show the passes between teams.
(PDF)

Figure S2 **Network diagram described by players as vertices connected by links representing passes in each 5-minute intervals in Kirin Cup 2006.** Black lines show the passes within each team, gray lines show the passes between teams.
(PDF)

Table S1
**Tests of power-law distributions against the exponential and the power law with cut-off distributions.** LR denotes the log-likelihood ratio under two competing distributions. Statistically significant 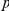-values are denoted in **bold**.(PDF)

Table S2 **Numbers of outgoing and incoming passes per player in World Cup 2006.**
(PDF)

Table S3 **Numbers of outgoing and incoming passes per player in Kirin Cup 2006.**
(PDF)

Text S1 **Evaluation of power-law distribution against other alternatives.**
(PDF)
