# Supplementary figures and images for: Common and Unique Network Dynamics in Football Games
Source: PLoS One. 2011 Dec 28;6(12):e29638. doi: 10.1371/journal.pone.0029638 (PMC3247158; doi:10.1371/journal.pone.0029638)

# World cup 06 Italy vs. France

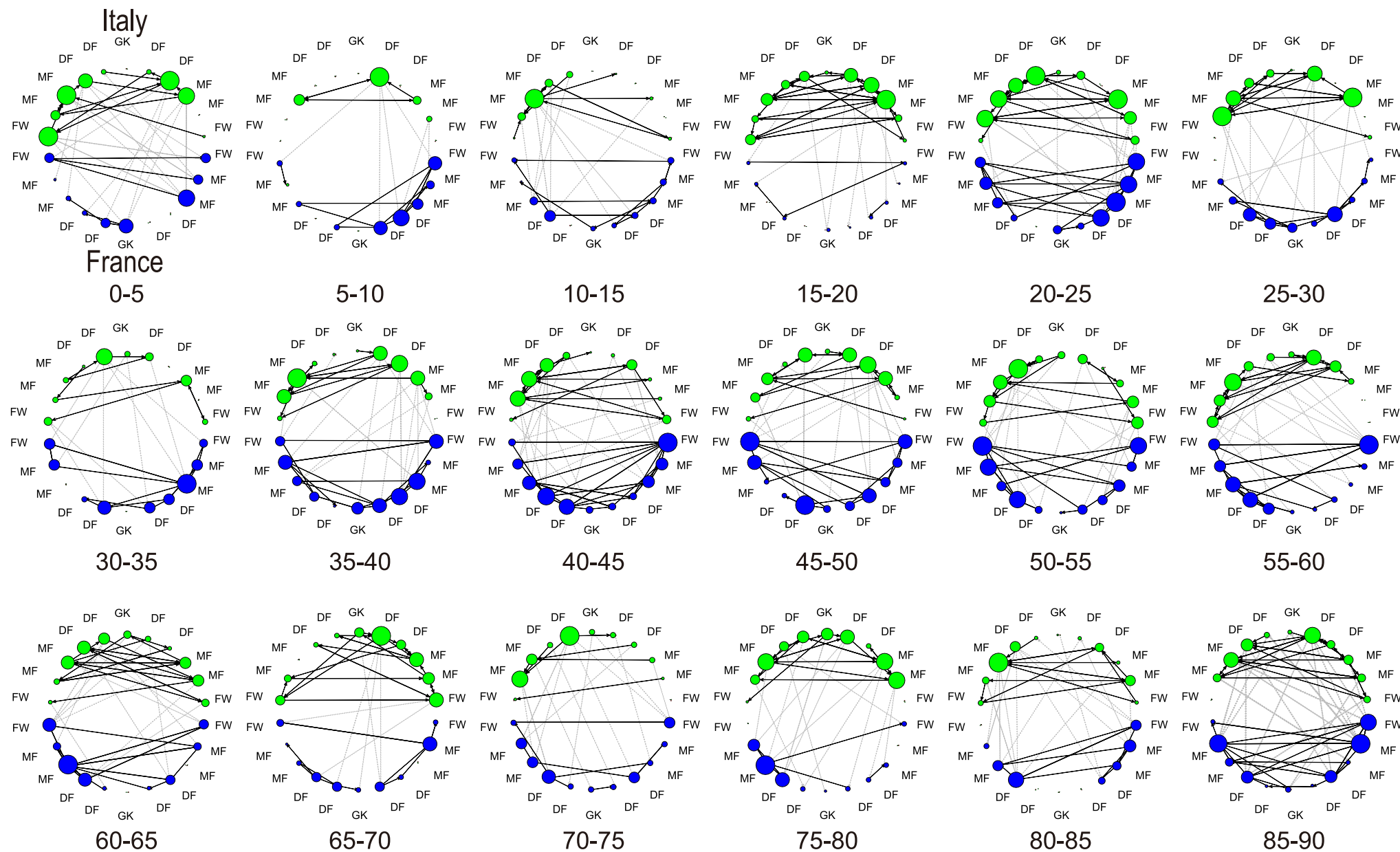

Supplement: Figure S1 — Network diagram described by players as vertices connected by links representing passes in each 5-minute intervals in World Cup 2006. Black lines show the passes within each team, gray lines show the passes between teams. (PDF) [file pone.0029638.s001.pdf]

## 2006 Kirin challenge cup Japan vs. Ghana

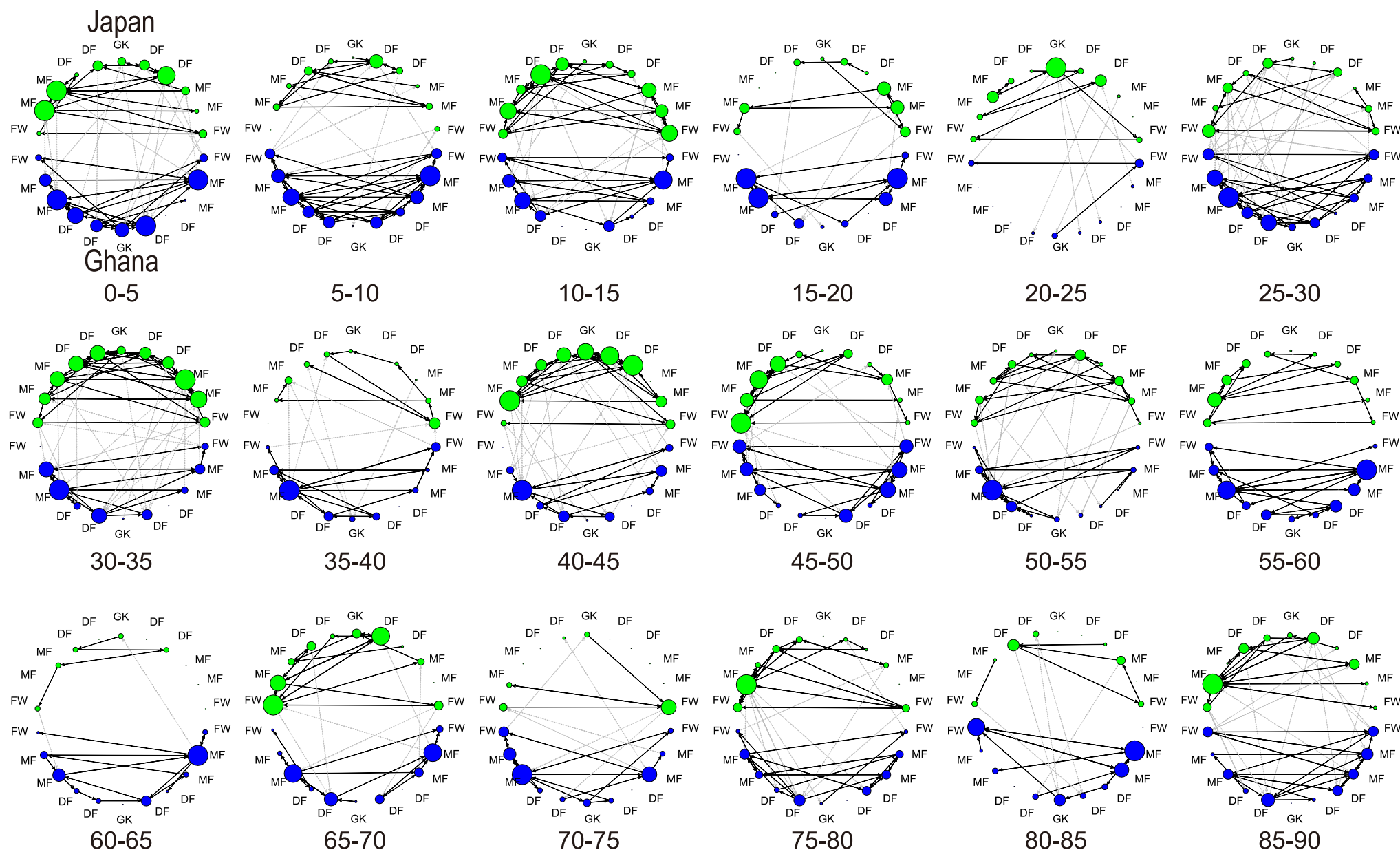

Supplement: Figure S2 — Network diagram described by players as vertices connected by links representing passes in each 5-minute intervals in Kirin Cup 2006. Black lines show the passes within each team, gray lines show the passes between teams. (PDF) [file pone.0029638.s002.pdf]
