# Supplementary material for: Common and Unique Network Dynamics in Football Games
Source: PLoS One. 2011 Dec 28;6(12):e29638. doi: 10.1371/journal.pone.0029638 (PMC3247158; doi:10.1371/journal.pone.0029638)
Supplement: Table S1 — Tests of power-law distributions against the exponential and the power law with cut-off distributions. LR denotes the log-likelihood ratio under two competing distributions. Statistically significant -values are denoted in bold. (PDF) [file pone.0029638.s003.pdf]

Table S 1: **Tests of power-law distributions against the exponential and the power law with cut-off distributions.** LR denotes the log-likelihood ratio under two competing distributions. Statistically significant p-values are denoted in **bold**.

2006 World cup			Power law p	Exponential LR	Exponential p	Power law+cut-off LR	Power law+cut-off p	Support for power law
Italy	1st	Out	0.08	-2.048	**0.04**	-3.512	**0.01**	with cut-off
		In	0.01	-2.593	**0.01**	-4.661	**0.00**	with cut-off
	2nd	Out	**0.51**	-0.126	0.90	-0.285	0.45	good
		In	0.00	-3.699	**0.00**	-6.940	**0.00**	with cut-off
France	1st	Out	0.05	-2.110	**0.03**	-3.134	**0.01**	with cut-off
		In	**0.23**	-1.700	0.09	-1.196	0.12	good
	2nd	Out	**0.32**	-0.921	0.36	-1.812	**0.06**	moderate
		In	0.09	-2.048	**0.04**	-2.570	**0.02**	with cut-off
2006 Kirin cup			p	LR	p	LR	p	
Japan	1st	Out	0.00	-4.159	**0.00**	-9.444	**0.00**	with cut-off
		In	**0.45**	-2.318	**0.02**	-2.689	**0.02**	moderate
	2nd	Out	**0.21**	-1.011	0.31	-1.833	**0.06**	moderate
		In	**0.19**	-1.305	0.19	-1.351	0.10	good
Ghana	1st	Out	0.03	-2.370	**0.02**	-1.778	**0.06**	none
		In	**0.39**	-1.330	0.18	-1.726	**0.06**	moderate
	2nd	Out	0.04	-2.720	**0.01**	-3.178	**0.01**	with cut-off
		In	0.00	-3.552	**0.00**	-7.917	**0.00**	with cut-off
