# Supplementary material for: Common and Unique Network Dynamics in Football Games
Source: PLoS One. 2011 Dec 28;6(12):e29638. doi: 10.1371/journal.pone.0029638 (PMC3247158; doi:10.1371/journal.pone.0029638)
Supplement: Table S2 — Numbers of outgoing and incoming passes per player in World Cup 2006. (PDF) [file pone.0029638.s004.pdf]

Table S 2: **Numbers of outgoing and incoming passes per player in World Cup 2006.**

Italy							France						
	1st half			2nd half				1st half			2nd half		
Pos.	Out	In	sum	Out	In	sum	Pos.	Out	In	sum	Out	In	sum
GK	3	2	5	11	5	16	GK	11	7	18	6	3	9
DF	11	12	23	19	14	33	DF	13	10	23	12	6	18
DF	15	10	25	17	10	27	DF	21	15	36	19	12	31
DF	13	13	26	20	18	38	DF	14	10	24	24	23	47
DF	22	15	37	12	10	22	DF	13	11	24	10	6	16
MF	29	31	60	28	23	51	MF	17	17	34	12	8	20
MF	3	4	7	18	16	34	MF	14	22	36	24	23	47
MF	17	23	40	13	21	34	MF	12	12	24	27	25	52
MF	23	19	42	23	22	45	MF	8	15	23	15	22	37
FW	6	8	14	5	15	20	FW	14	17	31	16	26	42
FW	5	10	15	5	17	22	FW	11	12	23	8	19	27
sum	147	147	294	171	171	342	sum	148	148	296	173	173	346
