# Supplementary material for: Common and Unique Network Dynamics in Football Games
Source: PLoS One. 2011 Dec 28;6(12):e29638. doi: 10.1371/journal.pone.0029638 (PMC3247158; doi:10.1371/journal.pone.0029638)
Supplement: Table S3 — Numbers of outgoing and incoming passes per player in Kirin Cup 2006. (PDF) [file pone.0029638.s005.pdf]

Table S 3: **Numbers of outgoing and incoming passes per player in Kirin Cup 2006.**

Japan							Ghana						
	1st half			2nd half				1st half			2nd half		
Pos.	Out	In	sum	Out	In	sum	Pos.	Out	In	sum	Out	In	sum
GK	15	9	24	12	6	18	GK	6	3	9	10	4	14
DF	21	18	39	9	4	13	DF	5	4	9	15	13	28
DF	20	20	40	17	17	34	DF	24	15	39	17	10	27
DF	13	12	25	12	12	24	DF	20	15	35	11	9	20
MF	23	19	42	10	9	19	DF	20	16	36	12	12	24
MF	18	17	35	20	13	33	MF	17	19	36	22	26	48
MF	24	27	51	27	30	57	MF	44	40	84	36	35	71
MF	22	18	40	1	5	6	MF	30	35	65	32	32	64
MF	20	22	42	19	21	40	MF	25	26	51	21	20	41
FW	6	15	21	14	21	35	FW	10	19	29	8	15	23
FW	15	20	35	10	13	23	FW	4	13	17	13	21	34
sum	197	197	394	151	151	302	sum	205	205	410	197	197	394
