# Supplementary material for: Common and Unique Network Dynamics in Football Games
Source: PLoS One. 2011 Dec 28;6(12):e29638. doi: 10.1371/journal.pone.0029638 (PMC3247158; doi:10.1371/journal.pone.0029638)
Supplement: Text S1 — Evaluation of power-law distribution against other alternatives. (PDF) [file pone.0029638.s006.pdf]

Supporting Information

Text S1.

Evaluation of power-law distribution against other alternatives

The power-law distributions were calculated and the power-law was compared with alternative hypotheses following the method for analyzing power-law distribution data proposed by Clauset et al. [1].

First, we estimated parameters x_{min} and γ of power-law model $x^{-\gamma}$. Then, we applied maximum likelihood fitting methods with goodness-of-fit tests based on the Kolmogorov-Smirnov (KS) statistics to our data for 1,000 replicates. If the resulting p-value was greater than 0.1, the power law was considered a plausible hypothesis for the data, otherwise it was rejected (see Table S1). We also compared the power law with exponential ($e^{-\lambda x}$) and power-law with cut-off ($x^{-\gamma}e^{-\lambda x}$) distributions as alternative hypotheses using the likelihood ratio test [2] to compute the likelihood of the data under two competing distributions and to make a quantitative judgment about whether the observed value was sufficiently far from zero. For each alternative, if the calculated p-value was small, i.e., $p < 0.1$, the sign was a reliable indicator of which model better fit the data (see Table S1).

In three cases, the power law appears to be truly convincing, in the sense that these are excellent fits to the data and none of the alternatives carries any weight: the outgoing link for Italy in the second half; the incoming link for France in the first half; and the incoming link for Japan in the second half. For the outgoing link for Ghana in the first half, the exponential distribution is favored over the power law. For the outgoing link for France in the second half, the outgoing link for Japan in the second half, and the incoming link for Ghana in the first half, because the p-values for the likelihood ratio tests for the exponential distribution are sufficiently large, the results of the tests are inconclusive. The other tests suggest that power law distributions are favored over the alternatives. Although the likelihood ratio test suggests that the exponential and power law with cut-off distributions could not ruled out, the p-value of the goodness-of-fit test for the incoming link for Japan in the first half was sufficiently large. Among the remaining cases, the power law with a cut-off is clearly favored over the pure power law.

We have used R and MATLAB software provided by Aaron Clauset (`http://tuvalu.santafe.edu/~aaronc/powerlaws/`).

References

1. Clauset A, Shalizi CR, Newman MEJ (2009) Power-law distributions in empirical data. SIAM Review 51: 661-703.

2. Vuong QH (1989) Likelihood ratio tests for model selection and non-nested hypotheses. Econometrica 57: 307-333.
